# Error in Results Section

**DOI:** 10.1001/jamanetworkopen.2020.31402

**Published:** 2020-12-04

In the Original Investigation titled “Association of Azithromycin Use With Cardiovascular Mortality,”^1^ published June 17, 2020, the hazard ratio data in the Results section for all-cause mortality and noncardiovascular death within 5 days were transposed. This article has been corrected.^1^
